# Treatment outcome measures for randomized controlled trials of antibiotic treatment for acute bacterial skin and skin structure infections in the emergency department setting

**DOI:** 10.1186/s12245-015-0060-9

**Published:** 2015-04-22

**Authors:** Michael Quirke, Abel Wakai

**Affiliations:** Emergency Care Research Unit (ECRU), Division of Population Health Sciences (PHS), Royal College of Surgeons in Ireland, 123 St. Stephen’s Green, Dublin 2, Ireland; Department of Emergency Medicine, Beaumont Hospital, Beaumont Rd, Dublin 9, Ireland

**Keywords:** Outcome measures, Cellulitis, Acute bacterial skin and skin structure infections, Randomized controlled trial

## Abstract

Acute bacterial skin and skin structure infections (ABSSSIs), which include cellulitis, abscesses, and wound infections, are among the most commonly encountered conditions in emergency departments (EDs) internationally. Primarily, as a result of the recent epidemic of community-associated methicillin resistant *Staphylococcus aureus* (CA-MRSA) in North America, ED attendances and hospital admissions secondary to ABSSSIs have increased significantly. First-line antibiotic drug therapies for ABSSSIs have therefore changed to take account of CA-MRSA and the threat of evolving antibiotic resistance. Prior to 2010, randomized controlled trials (RCTs) of antibiotic therapy for ABSSSI used broad trial inclusion criteria and utilized investigator-determined clinical resolution, 7 to 14 days after the end of therapy, as the primary outcome measure. In order to produce more objective, reproducible, and quantifiable primary outcome measures, the US Food and Drug Administration (FDA) Center for Drug Evaluation and Research and a multidisciplinary consortium convened by the Foundation for the National Institutes of Health (FNIH) issued significantly changed trial guidance criteria. The currently recommended primary outcome measure is an assessment of greater than 20% reduction in the area of erythema, edema, or induration from baseline, measured at 48 to 72 h after randomization and initiation of drug treatment. In contrast, the European Medicines Agency (EMA) still recommends measurement of clinical resolution at a later time period. We discuss the evolution of changes to trial guidance criteria issued by the FDA since 1998 and the potential difficulties of implementing the recommended primary outcome measured at an earlier time point in RCTs of outpatient antibiotic treatment performed in the ED setting.

## Review

### Introduction

Cellulitis, abscesses, and wound infections, most recently renamed as acute bacterial skin and skin structure infections (ABSSSIs) [1], represent a heterogeneous group of infections that are commonly encountered in clinical practice. In the emergency department (ED) setting, ABSSSIs account for between 1.5% and 3% of all attendances and, in a point prevalence survey of 12 European hospitals, were second only to respiratory tract infection as the most common cause for inpatient antibiotic therapy [2-5]. Despite this incidence data, there is a paucity of randomized controlled trial (RCT)-based evidence and recommendations to guide the management of ABSSSIs in the ED setting [6].

In the USA, community-associated methicillin-resistant *Staphylococcus aureus* (CA-MRSA) is the most common identifiable cause of purulent skin infections in EDs [7]. Data from the National Ambulatory Medical Care Survey showed that visits by patients with skin infections increased from 32.1 to 48.1 visits per 1,000 population between 1997 and 2005 [8]. Rather than substituting for methicillin-sensitive *S. aureus* as a primary cause of ABSSSI, the advent of CA-MRSA was associated with increased ED attendances [9] and hospital admissions [10] secondary to ABSSSI. CA-MRSA has now evolved to become a nosocomial pathogen with distinct genetic lineages causing infection in different hospital settings internationally [11,12]. CA-MRSA is not confined to North American EDs; a recent study showed that 22% of all purulent skin and soft tissue infections attending a Spanish ED in 2010 cultured CA-MRSA [13].

In addition to antibiotic stewardship and infection control policies, it follows that the identification of new drug therapies for ABSSSI is clinically important [14]. Equally important is the rationalization of existing antibiotic treatments for ABSSSIs, which are known to be heterogeneous [3,15], lack RCT-based evidence [6], and are associated with overtreatment of milder infections [15]. Whether to treat abscesses with concomitant antibiotics in addition to incision and drainage is also contentious, as most RCTs to date have been underpowered [16,17].

The need for more objective, reproducible, and reliable outcome measurements in RCTs of antibiotic treatment for ABSSSI prompted revision of clinical trial guidance criteria issued by the US Food and Drug Administration (FDA) [18]. Over the past several decades, the primary outcome measure of choice in clinical trials of antibiotic treatment for ABSSSI has been an investigator-determined clinical response, measured at 7 to 21 days after antibiotic treatment. Although this is clinically intuitive, it does not take account of factors which may influence response at a later time point such as host immune response and spontaneous lesion regression [19]. In addition, an objective and more reliable standard of measurement of the primary outcome would help ensure that evidence of superiority of a given treatment applies to ongoing and future trials of ABSSSI treatment (constancy assumption). Any new outcome measures would also have to be clinically relevant [18]. Patient-reported outcome measures or how a patient ‘feels, functions and survives’ have to date been significantly underreported in clinical trials of ABSSSI antibiotic therapy.

This review describes recommendations relevant to the ED setting, from regulatory authorities in the United States (US) and Europe concerning the measurement of treatment outcomes and inclusion criteria for RCTs of antibiotic therapy for ABSSSIs. Not only have there been changes to disease nomenclature, but also a complete revision of clinical trial entry criteria and the timing and method of measurement of the primary outcome.

### ABSSSI nomenclature

Nomenclature for this group of infections is confusing for two major reasons. Firstly, phenomenological descriptions of different types of infection (cellulitis, erysipelas, abscess), predisposing conditions (diabetic foot ulcer), eponymous diseases (Fournier’s gangrene, clostridial myonecrosis), and microbiological causes of infection [20,21] have resulted in heterogeneous terminology, much of which has only historical relevance. Secondly, repeated classifications by the US Food and Drug Administration (FDA) [1,22,23] and the Infectious Diseases Society of America (IDSA) [24,25], have resulted in inconsistencies in nomenclature. The term ABSSSI reflects the most current definition of the disease issued by the FDA for the purposes of performing and appraising RCTs [1].

### Previous FDA guidance

US regulations concerning the conduct of RCTs of antibiotic therapy for ABSSSI are issued in the form of ‘Guidance for Industry’ documents by the FDA Center for Drug Evaluation and Research (CDER). In the 1998 guidance document, infections were classified as complicated or uncomplicated [22] (Table 1).

**Table 1:** **Summary of relevant nomenclature issued by the US FDA and the Infectious Diseases Society of America (IDSA)**

**Source**	**Descriptor**	**Clinical conditions included**
FDA 1998 [22]	Uncomplicated skin and skin structure infection	Abscess, cellulitis, impetigo, furuncle
	Complicated skin and skin structure and soft tissue infection	Infections of deeper soft tissue or requiring significant surgical intervention (infected ulcers, burns, major abscess), or a significant underlying disease state that complicates response to treatment
FDA 2010 [23] and 2013 [1]	Acute bacterial skin and skin structure infections (ABSSSIs)	Bacterial infection of the skin with a minimum size of >75 cm^2^. Includes:
		•Cellulitis/erysipelas
		•Wound infection
		•Major cutaneous abscess
IDSA 2011 [24]	Purulent cellulitis	Cellulitis associated with purulent drainage or exudate in the absence of a drainable abscess
	Non-purulent cellulitis	Cellulitis with no purulent drainage or exudate and no associated abscess
IDSA 2014 [25]	Purulent skin and soft tissue infection (SSTI)	Abscess, furuncle, carbuncle, inflamed epidermoid cyst
	Non-purulent SSTI	Cellulitis, erysipelas, necrotizing fasciitis

Definitions for the inclusion of patients with infections were broad, leaving much to the discretion of clinical trial investigators. For example, there was no guidance for the measurement of minimum lesion size. Patients with a ‘major abscess’ were defined as having a ‘complicated’ infection but with no other discriminatory features; trial investigators undoubtedly enrolled a wide variety of major and ‘minor’ infections [26]. There were no clear restrictions to the number of patients enrolled with abscess, which became problematic in the context of the CA-MRSA epidemic in the US, with more patients enrolled from EDs with purulent skin and soft tissue infections (SSTIs) [26,27]. The primary outcome measure was ‘clinical cure’, interpreted as the resolution of signs and symptoms based on physician’s observation, need for alternative therapy, and patient comments [18]. Although intuitive, and still the primary outcome measure of choice in Europe, more objective, measurable, and reproducible outcome measures were required [28].

In 2010, the FDA published new draft guidance [23]. Two major changes to both the timing and assessment of the primary outcome were recommended. Firstly, the FDA guidance recommended changing the timing of primary outcome measurement from 7 to 21 days after the end of antibiotic treatment to an earlier measurement within 48 to 72 h of treatment commencement. Secondly, the new primary outcome recommended measuring cessation of the spread of lesion erythema, edema, or induration, in addition to resolution of fever, whereas previous guidance recommended investigator-determined clinical response or ‘cure’. In arriving at this new primary outcome measure, the FDA was guided by historical evidence from two trials of antimicrobial therapy for ABSSSI. In 1937, Snodgrass and Anderson performed two controlled trials comparing sulphanilamide and prontosil (a pro-drug of sulphanilamide) with antitoxin and UV light [29,30]. After 48 to 72 h of treatment, they recorded significant cessation in lesion spread and normalization of body temperature in the group receiving antibiotic treatment. Given that these two trials demonstrated historical evidence of sensitivity to drug effects (HESDE), the FDA recommended this new early primary outcome measure and as a secondary outcome, sustained clinical response at the later test-of-cure visit.

However, numerous authors have questioned the validity of using non-verifiable data obtained almost eight decades ago [26,31,32]. Over half of patients receiving sulphanilamide and 39% receiving ultraviolet (UV) light improved on day zero making the timing of treatment effect questionable. Furthermore, the constancy assumption (that superiority shown in historical trials still applies today) may not apply due to advances in medical care in the intervening years [28].

There was considerable controversy regarding the utilization of cessation of lesion spread as the primary outcome measure. Perhaps most significantly, cessation of lesion spread indicates that the patient is on a path to recovery rather than representing treatment success [26,31]. However, measurement of clinical response at an earlier time point is clinically intuitive. It is also more likely to represent an actual treatment effect, as assessing cure at a later time point introduces the confounding influences of host immune response and natural regression of the infection [19]. Evidence from recent trials supports early measurement of clinical response. In the ESTABLISH-2 study, the majority (>80%) of cases of ABSSSI showed early clinical response, which was sustained at the later assessment (0.7% to 3.8% of initial responders were deemed to have an ongoing infection at the later test-of-cure assessment) [33].

Given these controversies, the FDA commissioned a multidisciplinary report from the Foundation for the National Institutes of Health (FNIH) Biomarkers Consortium. This consortium analyzed both historical data and data from recently published trials which examined this new efficacy endpoint in conjunction with the older endpoint of ‘clinical cure’.

### Current FDA guidance

#### Definitions of ABSSSI

ABSSSIs are defined as cellulitis or erysipelas, wound infections, and abscesses of at least 75 cm^2^ measured by the area of redness, edema, or induration [1]. Measurement of the area of erythema has shown high inter-observer reliability when using a plastic ruler [34], but other methods such as planimetry and digital photography are poorly tested [18]. It is still unclear whether lesion diameter dictates infection severity [26]. In addition, there are currently no recommendations for measuring lesion size in pediatric patients, small adults, or specific body parts such as the face, hand, and perineum.

#### Clinical trial design, populations, and entry criteria

A mixture of the different ABSSSI disease entities should be included with a cap of 30% on the number of major abscesses enrolled [1]. The 2013 guidance does not specify that elevated body temperature is a requirement for enrollment. This contrasts with 2010 guidance which required four-hourly measurement of patient body temperature [23]. Two recent trials have shown that only 18 to 34% of patients with complicated ABSSSI have fever at baseline [35,36]. Requirement for elevated body temperature at enrollment could potentially exclude elderly patients, diabetics, and the immunocompromised, leading some authors to question whether fever is even on the causal pathway of the disease [18].

Limiting the number of patients with abscess to less than 30% of trial participants will minimize the bias that surgical treatment of abscesses may introduce to the RCT, and prevent overrepresentation of abscesses from regions where purulent cellulitis is more commonly encountered [1]. The role of adjunctive antibiotic therapy for abscess is contentious; since the majority of abscesses can be cured with incision and drainage alone [37,38], and many previous trials included smaller abscesses [31], very large sample sizes are required to show small differences in response rates [39].

#### Prior antibacterial drug therapy

Patients should ideally not have received any antibiotic therapy prior to trial enrollment unless there is objective evidence of the ABSSSI deteriorating while on treatment [1]. However, excluding all patients who previously received treatment would result in a population with a less severe illness (bias towards non-inferiority) and limit the external validity of the trial results. To address this, the FDA suggests ‘pragmatic’ approaches including prompt enrollment procedures and allowing less than 25% of the total population enrolled to have received a single dose of a short-acting antibiotic [1]. However, in the clinical setting, delaying the first dose of antibiotic to a patient while awaiting trial enrollment and randomization may not represent an acceptable standard of care and may be unethical. Since many patients attending EDs have already commenced oral antibiotic treatment, particularly in areas with developed primary care services, it will also be challenging to document objective clinical progression when the patient is being treated by a different practitioner.

#### Measurement of primary outcome measure

The primary outcome measure should assess for 20% or greater reduction in lesion size when measured at 48 to 72 h compared to baseline [1]. The timing and nature of this outcome measure is supported by evidence from recent RCTs [33,36,40], which demonstrate the feasibility of measurement at this time point. Further advantages of early assessment of the primary outcome include early identification of treatment failure, earlier de-escalation of broad-spectrum antibiotics, and possibly, earlier discharge on oral therapy [41].

#### Measurement of secondary outcome measures

According to the most recent FDA guidance, the secondary outcome measure should be investigator-determined clinical resolution measured between 7 and 14 days after the completion of therapy [1]. Measurement of clinical resolution at a specified time point after the end of treatment is important to provide evidence of a sustained effect and longer-term patient-reported outcome measures (PROMs) [18].

Measurement of reduction in the area of erythema represents a more objective, quantifiable, and reproducible primary outcome assessment than clinical cure at the end of therapy. However, there is still controversy regarding what constitutes a clinically meaningful trial outcome measure for RCTs of ABSSSI treatment [18,19,31]. A reduction in the area of erythema may not have any meaningful impact on how a patient functions or feels [18]. Previous FDA guidance was criticized for measuring cessation in lesion spread as the primary outcome measure, which may not, in reality, translate to a clinically successful treatment for patient or assessing physician [31]. Assessment of PROMs in RCTs of drug therapy for ABSSSI is overall lacking and deserving of further study.

It is worth noting that although the FDA issues guidelines pertaining to clinical trial design, the guidelines are not binding but represent ‘current thinking’ on issues related to trial design (Table 2). Several recent trials of ABSSSI antibiotic treatment have incorporated recommended outcome measures [33,36,42,43], including more recently PROMs [33].

**Table 2:** **Summary of FDA guidance document issued in 2013 and potential issues in implementation**

**FDA guidance 2013**	**Potential issues**
Definitions of ABSSSI	
Cellulitis or erysipelas, wound infections, and abscesses with at least 75 cm^2^ area of redness, edema, or induration.	Lesion size >75 cm^2^ is arbitrary [26].
Minimum lesion size of 75 cm^2^ differentiates ‘minor’ cutaneous abscess from ‘major’ abscess.	Minimum lesion size may exclude key groups (pediatric, small adults) and body parts (face, hand, genitalia) [28].
	Measurement of induration and edema is subjective [28].
	Depth of involvement may be more important than diameter [26].
Clinical trial design, populations, and entry criteria	
Mixture of ABSSSI entities.	Conflicting evidence regarding the efficacy of drug therapy for ‘minor’ abscess.
Limit on number of major abscesses to ≤30% of total enrolled.	
No requirement for elevated body temperature at enrollment.	Abscess less common in European ED settings than in North America due to lower prevalence of CA-MRSA.
Prior antibacterial drug therapy	
Encourage prompt enrollment procedures.	Prompt enrollment is difficult to achieve in many ED settings.
Allow enrollment of small number of patients who have received a single dose of a short-acting antibacterial drug within the previous 24 h.	Many ED patients have commenced therapy on presentation in areas with developed primary care services.
Allow enrollment of patients with objective documentation of clinical progression while on antibacterial therapy (i.e. not based on history alone).	Difficult to obtain objective evidence of clinical progression of previously treated ABSSSI, where treatment commenced in primary care.
Outcome measures and timing of assessments	
Primary outcome measure of lesion response at 48 to 72 h (≥20% reduction in lesion size when measured compared to baseline).	Achieving reliable endpoint measurement in trials of outpatient oral therapy may be difficult in ED setting, with higher loss to follow-up rate.
Secondary efficacy endpoint is a resolution of ABSSSI 7 to 14 days after completion of therapy.	No evidence that reduction in the size of lesion represents how patients feel or function [28].

### European Medicines Agency (EMA) guidance

The primary outcome measure recommended by the EMA is an investigator-determined clinical outcome (cure, failure, or indeterminate responses) measured at 7 to 14 days after the end of therapy. The EMA primary outcome measure is the same as the FDA-recommended secondary outcome measure [1]. Provided sponsors capture both early and late outcomes in multinational trials, separate analyses for US and European authorities are possible, and the same study could satisfy both sets of requirements [18]. It is likely that the development of two separate clinical development programs will incur significant costs to trial sponsors [31].

#### ED outcome measures for RCTs of antibiotic treatment for ABSSSIs

The feasibility of employing the recent FDA-recommended primary efficacy outcome measure (reduction in lesion size 48 to 72 h after randomization to treatment) may be challenging in ED-based RCTs of antibiotic treatment for ABSSSIs. ED-specific challenges to patient recruitment such as overcrowding and loss to follow-up of recruited patients who may not live in the ED’s catchment region are well recognized [44]. In an ED-based RCT for uncomplicated ABSSIs in children, in which trial participants were assessed by either a review visit or a phone call between 2 and 3 days post-enrollment, 35% to 38% of patients were followed up by a phone call [45]. This indicates potential difficulties in achieving objective measurement of the current FDA-recommended primary outcome measure in the ED setting [1].

Other trials of outpatient antibiotic therapy for ABSSSIs performed in the ED-setting have used investigator-determined clinical resolution of disease at the test-of-cure visit [9,46-49]. Although this latter primary outcome measure is less challenging to use in the ED setting, it can now be considered to have been superseded by the more recent FDA-recommended primary outcome measure (reduction in lesion size 48 to 72 h after randomization to treatment). Aside from requiring a considerably greater financial investment in terms of achieving day 3 follow-up, innovative measures of patient follow-up should be considered in future ED-based RCTs of outpatient antibiotic treatment for ABSSSIs to overcome some of the current challenges. For example, future ED-based RCTs could examine the feasibility of using telemedicine (including handheld devices such as mobile phones or tablet computers) or web-based platforms for trial participant follow-up.

## Conclusions

In the context of the recently updated 2013 FDA guidance regarding the conduct of RCTs of antibiotic treatment for ABSSSIs, it is timely for emergency medicine professional organizations, such as the Society for Academic Emergency Medicine (SAEM) in the US and the European Society for Emergency Medicine (EuSEM), to develop consensus recommendations to guide emergency medicine researchers on which outcome measures to use for ED-based RCTs of antibiotic therapy for ABSSSIs. In the meantime, emergency medicine researchers should use the primary outcome measure of reduction in lesion size between 2 and 3 days after randomization and clinical cure 7 to 14 days post end of therapy for ED-based RCTs of antibiotic treatment for ABSSSIs.

## Abbreviations

ABSSSI: Acute bacterial skin and skin structure infection
SSTI: Skin and soft tissue infection
ED: Emergency department
PROM: Patient-reported outcome measure
FDA: Food and Drug Administration
EMA: European Medicines Agency
FNIH: Foundation for the National Institutes of Health
CA-MRSA: Community-associated methicillin-resistant *Staphylococcus aureus*
IDSA: Infectious Diseases Society of America
SAEM: Society for Academic Emergency Medicine
EuSEM: European Society for Emergency Medicine
